# Long-Chain Acylcarnitines and Cardiac Excitation-Contraction Coupling: Links to Arrhythmias

**DOI:** 10.3389/fphys.2020.577856

**Published:** 2020-09-11

**Authors:** Hamish M. Aitken-Buck, Julia Krause, Tanja Zeller, Peter P. Jones, Regis R. Lamberts

**Affiliations:** ^1^Department of Physiology, School of Biomedical Sciences, University of Otago, Dunedin, New Zealand; ^2^University Heart and Vascular Center, University Medical Center Hamburg-Eppendorf, Hamburg, Germany; ^3^German Center for Cardiovascular Research (DZHK), Partner Site Hamburg, Hamburg, Germany

**Keywords:** long-chain acylcarnitines, metabolomics, cardiac pathophysiology, electrophysiology, arrhythmias, excitation-contraction coupling, calcium

## Abstract

A growing number of metabolomic studies have associated high circulating levels of the amphiphilic fatty acid metabolites, long-chain acylcarnitines (LCACs), with cardiovascular disease (CVD) risk. These studies show that plasma LCAC levels can be correlated with the stage and severity of CVD and with indices of cardiac hypertrophy and ventricular function. Complementing these recent clinical associations is an extensive body of basic research that stems mostly from the twentieth century. These works, performed in cardiomyocyte and multicellular preparations from animal and cell models, highlight stereotypical derangements in cardiac electrophysiology induced by exogenous LCAC treatment that promote arrhythmic muscle behavior. In many cases, this is coupled with acute inotropic modulation; however, whether LCACs increase or decrease contractility is inconclusive. Linked to the electromechanical alterations induced by LCAC exposure is an array of effects on cardiac excitation-contraction coupling mechanisms that overload the cardiomyocyte cytosol with Na^+^ and Ca^2+^ ions. The aim of this review is to revisit this age-old literature and collate it with recent findings to provide a pathophysiological context for the growing body of metabolomic association studies that link circulating LCACs with CVD.

## Introduction

Metabolomic studies have identified numerous circulating metabolites that correspond to an enhanced risk of cardiovascular disease (CVD) (McGarrah et al., 2018). One of the most commonly implicated groups of metabolites are long-chain acylcarnitines (LCACs, acylcarnitines with ≥ 14 carbon atoms) (Ruiz-Canela et al., 2017). LCACs are intermediates of intracellular fatty acid metabolism. High LCAC levels have been associated with key CVDs, including heart failure (HF), coronary artery disease (CAD), and cardiac arrhythmias (Shah et al., 2010; Kalim et al., 2013; Rizza et al., 2014; Zordoky et al., 2015; Krause et al., 2018; Verdonschot et al., 2019). Moreover, the LCAC-CVD association has been reported to be proportional to disease severity in many studies (Lai et al., 2014; Ahmad et al., 2016). The potential for therapeutic outcomes from derived from cardiovascular metabolomic associations is dependent on understanding how specific metabolites affect heart function. Therefore, due to the growing clinical interest in LCACs, it is pertinent to appraise what is known about LCACs in cardiac pathophysiology.

LCACs accumulate intracellularly and embed within membrane compartments of the ischemic myocardium (Idell-Wenger et al., 1978; Liedtke et al., 1978; Lopaschuk et al., 1988; Corr et al., 1989). Additionally, inhibition of LCAC biogenesis has been reported to attenuate arrhythmic and contractile deficits that follow ischemia, suggesting a pathophysiological role of the metabolites in cardiac dysfunction (Knabb et al., 1986; Corr et al., 1989). Subsequent studies of exogenous LCAC superfusion in experimental models have recapitulated these manifestations of cardiac dysfunction (Corr et al., 1981; Sakata et al., 1989; Meszaros and Pappano, 1990; Roussel et al., 2015); however, there are key controversies and limitations that hinder the understanding of the LCACs as cardiac effectors. The aim of this review is to collate what is currently known about LCACs as effectors of cardiac function and to provide a pathophysiological basis for the recent metabolomic interest of circulating LCACs in the plasma of CVD patients. Specifically, this will include discussion of LCAC effects on myocardial electrophysiology, contractility, and arrhythmias, as well as the underlying excitation-contraction coupling modulations. For clarity, we refer to LCACs as a general acylcarnitine class throughout this review; if specific LCAC species (such as palmitoylcarnitine) are discussed this will be stated.

### LCAC Biogenesis and Cardiovascular Disease Associations

**FIGURE 1:**
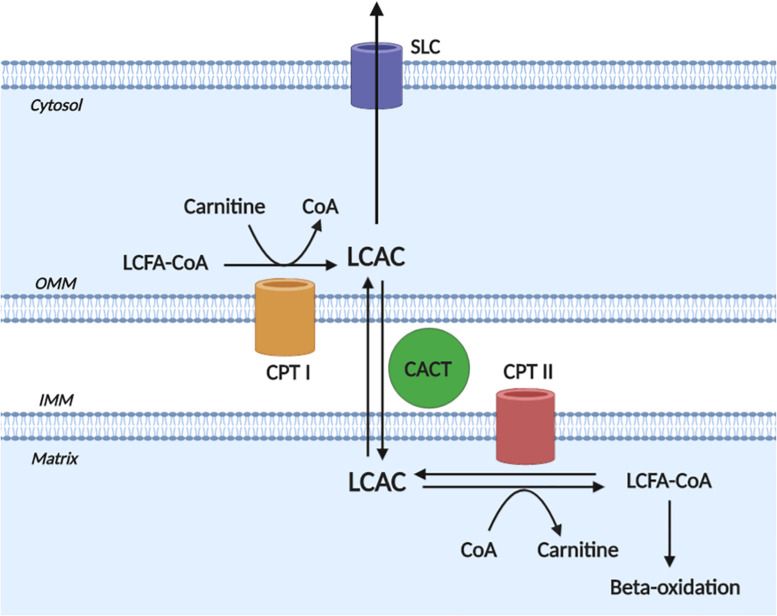
Long-chain acylcarnitine biogenesis. Long-chain acylcarnitines (LCACs) are the product of long-chain fatty acyl-CoA (LCFA-CoA) esterification to a free cytosolic carnitine, which is catalyzed by carnitine O-palmitoyltransferase I (CPT I) at the outer mitochondrial membrane (OMM). LCAC translocation across the inner mitochondrial membrane (IMM) and into the mitochondrial matrix occurs via the action of carnitine acylcarnitine translocase (CACT). Once in the matrix, the LCAC genesis reaction is reversed by carnitine O-palmitoyltransferase II (CPT II) to yield the constituent LCFA-CoA and carnitine. The LCFA-CoA can then continue to oxidative metabolism via β-oxidation. Note, that the reactions of CPT II and CACT are bi-directionally catalytic and cytosolic LCACs can be exported, presumably via a member of the solute carrier family (SLC). Figure created using BioRender.com.

A detailed description of LCAC biogenesis is beyond the scope of this review and has been done previously (Lopaschuk et al., 2010; Reuter and Evans, 2012); however, to appreciate the pathophysiological effects of LCACs, an understanding of LCAC origin is required. Briefly, LCACs are generated as the product of LCFA CoA esterification with L-carnitine by carnitine O-palmitoyltransferase (CPT) I (CPT I) (Figure 1). LCACs primarily function to traverse the mitochondrial membranes, which are impermeable to LCFA-CoA molecules. This LCAC translocation allows LCFA-CoAs to be re-generated within the mitochondrial matrix and to enter β-oxidation. The LCAC translocation process is catalyzed by the carnitine-acylcarnitine translocase (CACT), while the conversion of LCACs back to LCFA-CoA is dependent on CPT II at the inner mitochondrial membrane. CACT and CPT II are bi-directionally catalytic, which means that LCACs can be generated within the mitochondria and exported into the cytosol and, eventually, into the circulation (Noland et al., 2009; Makrecka-Kuka et al., 2017). Cardiac muscle has a relatively high LCAC content due to the preference of LCFAs for oxidative metabolism (Lopaschuk et al., 2010; Makrecka-Kuka et al., 2017). Furthermore, myocardial LCAC content is positively correlated with plasma LCAC concentrations – a feature that remains proportional during fasting, metabolic challenge, or in CVD (Lai et al., 2014; Makrecka et al., 2014; Makrecka-Kuka et al., 2017). Plasma LCAC concentrations have been estimated to range from ∼0.4 up to 3 μM, the most abundant of which are palmitoylcarnitine and oleoylcarnitine (Costa et al., 1997; Chace et al., 2001; Reuter et al., 2005; Ramos-Roman et al., 2012). However, in inherited fatty acid oxidation disorders that manifest with cardiac dysfunction (among other symptoms), such as CPT II deficiency, extreme plasma LCAC upregulation has been reported (>40 μM) (Chace et al., 2001; McHugh et al., 2011).

A growing body of evidence has correlated circulating LCACs with total CVD risk and CVD prognosis (Kalim et al., 2013; Cheng et al., 2015; Ruiz-Canela et al., 2017). LCACs have been independently associated with HF and indices of left ventricle (LV) remodeling and function in a manner proportional to disease stage and severity (Lai et al., 2014; Rizza et al., 2014; Zordoky et al., 2015; Ahmad et al., 2016; Hunter et al., 2016; Lanfear et al., 2017; Ruiz et al., 2017; Elmariah et al., 2018; Verdonschot et al., 2019). In some epidemiological cohorts, LCAC levels are reported to be increased in HF with reduced ejection fraction (HFrEF) relative to either non-failing controls or patients with HF with preserved ejection fraction (HFpEF) (Ahmad et al., 2016; Hunter et al., 2016; Lanfear et al., 2017; Verdonschot et al., 2019). Others have found a greater level in early stage HFpEF relative to HFrEF; however, this may be due to discrepancies in HFrEF severity and cohort sizes (Zordoky et al., 2015; Bedi et al., 2016). Significant circulating LCAC upregulation occurs in both ischemic and non-ischaemic HF etiologies, although the upregulation is significantly greater in ischaemic HF patients (Lanfear et al., 2017; Verdonschot et al., 2019). Importantly, circulating LCAC levels were found to normalize to non-failing levels following left ventricular assist device implantation of aortic valve replacement (Ahmad et al., 2016; Elmariah et al., 2018). In addition to HF, studies of the CATHGEN plasma biorepository have found significant associations of LCACs and CAD risk and mortality (Shah et al., 2010, 2012). Furthermore, from the same biorepository, high blood LCAC levels have been associated with pulmonary hypertension (Luo et al., 2017). There is currently a paucity of clinical investigation of the LCAC interaction with cardiac arrhythmias; however, preliminary findings from a large patient cohort indicate a significant link between serum LCACs and atrial fibrillation risk (Krause et al., 2018).

Taken together, metabolomics has provided compelling evidence for an independent association of circulating LCACs with ischaemic and non-ischaemic CVD risk and severity. To date, determination of blood LCAC levels has both diagnostic and prognostic value for manifestations of HF and CAD, and there is promising evidence for a similar LCAC association with cardiac arrhythmias. The following sections detail what is understood about the pathophysiological effects of LCACs on cardiac electrophysiology and contractility.

### LCAC Effects on Cardiac Electrophysiology and Arrhythmias

**FIGURE 2:**
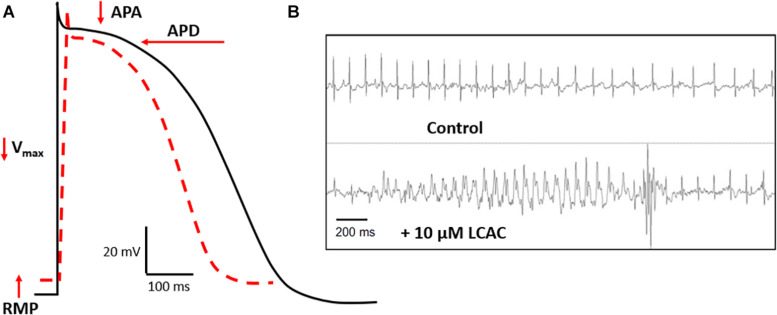
LCAC effects of electrophysiology and arrhythmia. **(A)** Long-chain acylcarnitines (LCACs) stereotypically alter the cardiac action potential (AP). Normal ventricular AP schematic represented in black, LCAC-altered AP in red hatched line. Characteristic effects of LCACs include reductions in AP duration (APD), amplitude (APA), the maximal rate of depolarization (V_*max*_), and a depolarization of the resting membrane potential (RMP). These AP alterations predispose cardiac muscle to arrhythmias. **(B)** Representative trace from echocardiogram of a mouse model infused with 10 μM LCAC (palmitoylcarnitine). Relative to control trace, observable electrical arrhythmia occurred with LCAC infusion, notable are premature ventricular complexes. Figure reprinted from Roussel et al. (2015), with permission from Elsevier.

Complimenting these recent clinical findings is literature detailing the effects of LCACs on cardiac function. In contrast with the modernity of CVD metabolomics, much of the translatable basic research is age-old and based in specific animal models. LCAC exposure has been reported to alter cardiac electrophysiological and arrhythmia susceptibility stereotypically (Corr et al., 1984; DaTorre et al., 1991). As shown in Figure 2, this includes reductions in the amplitude and duration of cardiac action potentials (APA and APD, respectively), as well as a decreased rate of depolarization (V_*max*_ of phase 0) and, often, a depolarization of the resting membrane potential (RMP). Furthermore, these alterations can establish a substrate for electrical arrhythmias (Knabb et al., 1986; Corr et al., 1989; Sakata et al., 1989; Meszaros and Pappano, 1990).

Relative to normoxic conditions, total LCAC content has been found to increase 3- to 70-fold in the ischaemic mammalian myocardium depending on the myocardial compartment and hypoxia duration/severity (Idell-Wenger et al., 1978; Liedtke et al., 1978; Knabb et al., 1986; Corr et al., 1989; McHowat et al., 1993). Within the mammalian myocardium, ischemia acutely (within minutes) reduces the APA and APD, V_*max*_, and RMP, leading to a predisposition for ventricular complexes and fibrillations (Corr et al., 1978; Pogwizd and Corr, 1987). These *in vivo* action potential and arrhythmic derangement have been recapitulated *in vitro* using myocyte cultures, in multicellular preparations, and *in situ* (Knabb et al., 1986; Corr et al., 1989; Yamada et al., 1994). Importantly, in these experimental models, pre-hypoxic treatment with sodium 2-[5-(4-chlorophenyl)-pentyl]-oxirane-2-carboxylate (POCA) or oxfenicine, both inhibitors of CPT I, prevents membrane and cytosolic LCAC accumulation in early ischaemia and the genesis of electrophysiological derangements (Knabb et al., 1986; Corr et al., 1989; Yamada et al., 1994). Many have reported a reversibility of LCAC-mediated effects in ischaemia following normoxic reperfusion, suggesting direct enzymatic or ionic modulation by LCACs (Corr et al., 1989). These early reports clearly implicate endogenous LCAC upregulation in early ischaemic arrhythmogenesis, which is relevant to recent metabolomic associations of LCAC species with ischaemic CVD (Lai et al., 2014; Ahmad et al., 2016; Ruiz et al., 2017).

First described by Corr et al. (1981), exogenous LCAC application was found to reduce the APA, APD, V_*max*_, and RMP dose-dependently (75–300 μM) and with an effect latency analogous to that found in the ischaemic myocardium (2–10 min). These findings have been replicated by many groups in canine, guinea pig, and rabbit isolated cardiomyocyte and multicellular preparations exposed to similarly high LCAC doses and to lower LCAC doses (0.1–50 μM). Moreover, as with endogenous LCAC upregulation, exogenous LCAC delivery also induces spontaneous electrical arrhythmias, inhibits gap junction conductance, and promotes *in vivo* ventricular extra-systoles (see Figure 2B; Hayashi et al., 1982; Nakaya and Tohse, 1986; Aomine et al., 1988; Sakata et al., 1989; Meszaros and Pappano, 1990; Mèszàros, 1991; Wu and Corr, 1992, 1994, 1995; Sato et al., 1993; Shen and Pappano, 1995; Roussel et al., 2015). More recent work has found that LCAC-mediated APD retardation can be modeled *in silico* based on results in recombinant HEK-293 cells (Ferro et al., 2012). These electrical derangements associated with exogenous LCACs also exhibit reversibility; however, this is more common at lower doses, presumably due to detergent-like effects evident at high LCAC doses (Nakaya and Tohse, 1986; Busselen et al., 1988; Sakata et al., 1989). Others have reported less straight-forward LCAC effects. LCAC superfusion at a 1–10 μM range has been shown to increase the APD in guinea pig cardiomyocytes (Meszaros and Pappano, 1990; Xu and Rozanski, 1998); however, the effect was biphasic, progressing to an eventual APD decrease within 15 min of treatment (Meszaros and Pappano, 1990). Furthermore, APD modulation was suggested to occur only with intracellular LCAC delivery and not extracellular (Xu and Rozanski, 1998); although, this possible delivery discrepancy has been investigated by others who found a similar potency of LCAC effects irrespective of delivery site (Wu and Corr, 1992).

In summary, whether endogenously upregulated or applied exogenously, LCACs dose- and time-dependently alter cardiac electrophysiology and promote arrhythmic muscle behavior. Stereotypically, LCAC exposure depolarises the RMP, decreases the V_*max*_, and retards the APA and APD, while promoting muscle electrical automaticity and conduction deficits (Corr et al., 1984; DaTorre et al., 1991). These effects are consistent whether delivered via an intra- or extracellular method. Importantly, LCAC levels that induce electrophysiological derangements and arrhythmias are translatable to circulating levels in ischaemic and non-ischaemic CVD.

### LCAC Effects on Cardiac Contractility

In addition to extensively reproduced acute electrophysiological derangements induced by LCAC exposure, LCACs are also reported an effect on myocardial contractility. However, the direction of inotropic modulation induced by LCACs is inconclusive. Many have reported a positive inotropic effect, with exogenous LCAC concentrations as low as 1–10 μM reported to increase guinea pig papillary muscle tension by 15–100% dose-dependently (Clarke et al., 1996). Higher LCAC doses (30–300 μM), which are sufficient to induce electrical automaticity, augment this positive contractility effect to ∼200 and 600% of pre-exposure levels (Nakaya and Tohse, 1986; Aomine et al., 1988; Sakata et al., 1989; Clarke et al., 1996). LCAC superfusion has been found to increase isolated guinea pig myocyte shortening by 47 and 100% when administered at 1 and 3 μM doses, respectively (Shen and Pappano, 1995). Additionally, positive inotropy has been reported in avian ventricular muscle strip and beating myocyte aggregates treated with LCACs at 2–300 μM exogenous concentrations, indicating the effect has some consistency across species (Inoue and Pappano, 1983; Patmore et al., 1989). The latency of these positive contractility effects is inversely related to LCAC concentration. Inotropic modulations of multicellular preparations typically manifest within 10–20 min at higher exogenous doses (100–300 μM), while lower doses (1–30 μM) require relatively longer exposure durations ranging from 30 to 120 min (Inoue and Pappano, 1983; Nakaya and Tohse, 1986; Aomine et al., 1988; Sakata et al., 1989; Clarke et al., 1996). At equivalent lower doses, inotropic latency is reduced in isolated myocytes and myocyte aggregates (Patmore et al., 1989; Shen and Pappano, 1995), most likely due to a greater LCAC to membrane phospholipid mole ratio (discussed later).

Others have provided evidence for a negative contractility effect of LCACs. Accumulation of LCACs in ischaemia has been correlated with acute decreases in cat and pig *in vivo* LV function (Corr et al., 1989). Furthermore, CPT I inhibition with POCA during early ischaemia significantly attenuates this mechanical dysfunction in parallel with a prevented reduction in LCAC content (Corr et al., 1989; Yamada et al., 1994). Interestingly, genetic mouse models of heart-specific CPT I deletion exhibit significant left ventricular systolic dysfunction in response to pressure overload that exacerbates progressively with duration of CPT I gene knockout (He et al., 2012; Haynie et al., 2014). This *in vivo* mechanical deficit induced by chronic CPT I inhibition is paralleled by marked ventricular hypertrophy and an increased susceptibility to premature death (He et al., 2012). It should be noted, however, that controversy exists in the cardiac consequence of CPT I deletion, as others have reported attenuation of obesity associated left ventricular hypertrophy and contractile impairment when the genetic deletion is induced (Zhang et al., 2016). An additional report has linked similar structural remodeling and cardiac dysfunction in a myocardium-specific CPT II deficient mouse model (Pereyra et al., 2017). In this study, the ∼5-fold increase in left ventricular mass could not be ameliorated by classical hypertrophy attenuators, resulting in entire litter lethality at 26 weeks (Pereyra et al., 2017). The current paucity of CPT II genetic models and the controversial role of CPT I activity modulation in cardiac dysfunction (Abdurrachim et al., 2015), whether by acute pharmacological inhibition or chronic genetic deletion, contributes to the challenge of determining the efficacy of CPT I a therapeutic target in CVD, as will be discussed later (section “Research Gaps and Outlook”).

Exogenous LCAC application at concentrations of analogous to those found to increase papillary muscle contractility have been found to induce mechanical dysfunction in Langendorff-perfused of isolated rodent hearts. One group consistently reported that LCAC doses as low as 2.5 μM reduces left ventricular systolic pressures (Hara et al., 1996; Xiao et al., 1997), while at slightly higher doses (4–10 μM) both systolic and diastolic dysfunction is apparent (Hara et al., 1996, 1997; Arakawa et al., 1997; Xiao et al., 1997; Arakawa and Hara, 1999; Maruyama et al., 2000). Similarly, a separate group found that LCAC exposure of isolated rat hearts promotes irreversible ventricular contracture at a dose range of 1.6–9.7 μM (Busselen et al., 1988), while another group suggested that concentrations of 0.1–0.3 μM are sufficient to detrimentally increase ventricular diastolic tension (Criddle et al., 1990). A further study utilized papillary muscles from rabbits, finding an overall negative inotropic effect following 50 μM LCAC superfusion (Patel et al., 1999). The latency required for negative inotropy in isolated rat hearts is notably reduced relative to the equivalent lower (20–60 min for negative inotropy) and higher doses (5–15 min) that increase papillary muscle contractility (Busselen et al., 1988; Criddle et al., 1990; Hara et al., 1996, 1997; Arakawa et al., 1997; Xiao et al., 1997; Arakawa and Hara, 1999; Wu et al., 2009).

A biphasic inotropic effect has also been reported across a range of LCAC doses, species, and tissue preparations. Many have reported a transient increase precedes the sustained decline in ventricular pressure of isolated rat or rabbit hearts induced by 4–50 μM administration (Hara et al., 1996; Xiao et al., 1997; Patel et al., 1999; Maruyama et al., 2000). Similarly, positive inotropy induced in guinea pig papillary muscles, canine Purkinje fibers, and avian myocyte aggregates by higher LCAC doses (30–300 μM) often exhibits a progressive decline in force output following the initial force increase (Nakaya and Tohse, 1986; Patmore et al., 1989; Sakata et al., 1989; Clarke et al., 1996). Clinical metabolomic studies have identified associations of circulating LCACs and *in vivo* cardiac function measured in cardiac surgery patients (Elmariah et al., 2018). Several LCACs are significantly positively correlated with left ventricular mass and negatively with left ventricular ejection fraction (Elmariah et al., 2018). Furthermore, circulating LCAC species are negatively associated with 6 m walking distance in heart failure patients, which is a clinical surrogate of overall cardiovascular performance (Lanfear et al., 2017; Elmariah et al., 2018). These findings suggest a detrimental contractility effect of circulating LCACs on cardiac function. The basic literature, however, is less conclusive. Whether the inotropic effect of acute LCAC exposure is positive or negative appears to depend on the exogenous LCAC concentration and treatment duration, the tissue preparation, and the species used in the study. Positive inotropy has been almost exclusively reported in studies using papillary muscles from guinea pigs, while negative inotropy has been strongly argued from findings in Langendorff-perfused rat hearts.

This discrepancy may be due to differences between superfusion and perfusion delivery of the LCAC in papillary muscle and isolated heart preparations, respectively (Busselen et al., 1988). In addition, variation in the nutritional composition of the physiological solutions used to nourish the different myocardial preparations could influence contractility. For instance, inclusion of pyruvate in superfusion buffer of human multicellular myocardium preparations results in a potent inotropic effect (Hasenfuss et al., 2002). Importantly, all of the aforementioned studies of LCAC effects on cardiac contractility have utilized glucose-based superfusion and perfusion solutions, suggesting that the result discrepancy is not due to differential myocardial nourishment. However, the influence of differences in metabolic demand as well as oxygen and nutrient diffusion gradients between multicellular preparations and isolated working hearts cannot be excluded as a reason for the discrepancy. The compositional complexity of whole heart models relative to multicellular muscle strips could also be a reason for the inconclusive inotropic effect of LCACs. As will be discussed in more detail below, the amphiphilic nature of LCAC compounds permits cardiomyocyte membrane incorporation. In a multicellular muscle preparation, superfused exogenous LCACs have relatively less access to myocardial membranes when compared to LCACs perfused through the coronary circulation of isolated hearts. Moreover, a direct interaction of LCACs with the coronary vasculature cannot be ruled out as a variable that confounds the inotropic output of Langendorff-perfused hearts. The discrepancies in pre-clinical models indicate further study using *ex vivo* and *in vivo* human myocardium is required to more conclusively determine the inotropic effect of LCACs.

### Cardiac Excitation-Contraction Coupling Alterations Induced by LCACs

**FIGURE 3:**
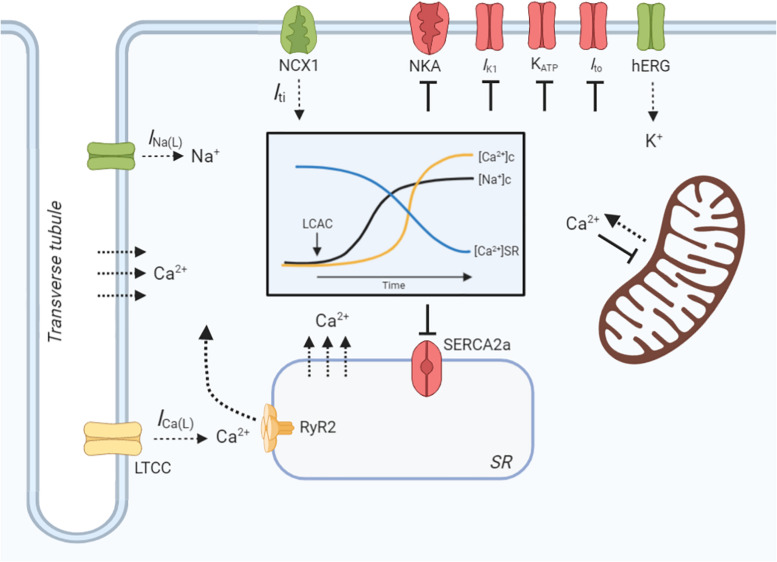
LCAC effects on cardiac excitation-contraction coupling. Schematic illustration of long-chain acylcarnitine (LCAC) effects on key cardiac excitation-contraction coupling proteins and compartment ion concentrations. Proteins or enzymes inhibited by LCACs are annotated as red, those that are stimulated are green, and those that are controversially affected by LCACs are in yellow. LCACs promote a sarcolemmal late Na^+^ current (I_*Na(L*__)_), which contributes to an increase in cytosolic Na^+^ concentration ([Na^+^]c, black line, inset figure). LCACs also inhibit the Na^+^/K^+^-ATPase (NKA), further increasing the [Na^+^]c. The cytosolic Ca^2+^ concentration ([Ca^2+^]c, yellow line, inset figure) is increased by LCAC application. This is contributed to by influx of Ca^2+^ from the extracellular fluid, either through the L-type Ca^2+^ channel (LTCC) or via membrane disruption; this is currently inconclusive. LCACs reduce the sarcoplasmic reticulum (SR) Ca^2+^ concentration ([Ca^2+^]SR, blue line, inset figure), thereby contributing to cytosolic Ca^2+^ overload. The dependence of ryanodine receptor (RyR2)-mediated SR Ca^2+^ release is unclear in cardiac cardiomyocytes. The sarcoendoplasmic reticulum Ca^2+^ ATPase (SERCA2a) is inhibited by LCACs, impairing Ca^2+^ removal from the cytosol. Ca^2+^ release from the mitochondria is stimulated by LCACs but uptake is inhibited. The I_*Na(L)*_ promoted by LCACs induces sarcolemmal Na^+^/Ca^2+^ exchange (NCX) via NCX1, which augments Ca^2+^ overload and stimulates a transient inward current (I_*ti*_) that provides the ionic substrate for spontaneous electrical activity. Ca^2+^ may also enter the cytosol from the extracellular environment or from the SR independently of protein facilitation, instead traversing the membranes due to amphipathic properties of LCACs. LCACs have been shown to inhibit the I_*K1*_, which contributes to resting membrane potential depolarization. Cardiac K_*ATP*_ channels and transient outward K^+^ current (I_*to*_) are inhibited by LCACs, thereby slowing repolarization. In contrast, LCACs stimulate the hERG K^+^ channels, resulting in enhanced repolarization and reduction of the action potential duration. Figure created using BioRender.com.

Cardiomyocyte contractility and electrophysiology, and the induction of arrhythmias, is dependent on a paradigm of ion-handling processes known as excitation-contraction coupling (ECC). LCACs have previously been investigated as modulators of many key enzymatic and channel ion fluxes. The pleiotropy of effects spans mechanisms that alter myocyte Ca^2+^, Na^+^, and K^+^-handling (Figure 3). These effects will be reviewed in the following sections; however, a prefacing discussion of the amphiphilic nature of LCACs and their membrane perturbing effects is necessary.

#### Membrane Perturbing Effects of LCACs

LCAC membrane incorporation has been modeled in purified phospholipid vesicles, with findings suggesting that LCACs alter the biophysical properties of cardiomyocyte membranes in a manner that could be linked to electrophysiological derangements (Fink and Gross, 1984; Requero et al., 1995a, b). LCACs embed within membrane bilayers and increase membrane fluidity in proportion to the LCAC to phospholipid mole ratio (Fink and Gross, 1984; Requero et al., 1995a, b). Requero et al. (1995a) found that LCAC membrane saturation eventually induces vesicle leakage and complete solubilization, indicating that LCACs act like surfactant compounds within cell membranes that disrupt the packing of adjacent phospholipids. It should be noted, however, that at a LCAC to phospholipid ratio sufficient to induce membrane leakage, significant incubation time is required (time required for 50% leakage was 2.6 h) that markedly exceeds that reportedly needed to induce electrophysiological and contractility effects (Requero et al., 1995a).

Further evidence supports LCAC-mediated membrane perturbation as a dependent mechanism in LCAC effects on cardiac function. Firstly, as will be discussed below, LCACs induce Ca^2+^ overload within cardiomyocytes via release of sarcoplasmic reticulum (SR) Ca^2+^ and inhibition of mitochondrial Ca^2+^ uptake (Piper et al., 1984; El-Hayek et al., 1993; Yamada et al., 2000). When investigated, this Ca^2+^ overload effect has been shown to depend on LCAC fatty acyl chain length, suggesting that longer LCACs have greater membrane penetration and, therefore, cause greater membrane disruption (Piper et al., 1984; El-Hayek et al., 1993; Yamada et al., 2000). Furthermore, LCAC binding to phospholipids within membrane vesicles is stronger for LCACs with longer fatty acyl chains (Requero et al., 1995b). Secondly, when canine Purkinje fibers are co-treated with LCACs and another metabolite, lysophosphatidylcholine, the combined electrophysiological effects of the amphiphiles are analogous to those induced by comparable concentrations of each metabolite alone (Corr et al., 1981). This additive rather than synergistic effect of the different amphiphiles suggests membrane incorporation alters cardiomyocyte function instead of a direct ion channel or enzymatic interaction. Finally, isolated rat hearts perfused with LCACs were shown by Busselen et al. (1988) to release myoglobin into the coronary effluent in a dose- and time-dependent manner. The markedly elevated myoglobin content present following acute LCAC exposure suggests that sarcolemma disruption was responsible for the significant negative inotropic effect observed in the rat hearts (Busselen et al., 1988).

Opposing these findings of membrane perturbation and potentially irreversible sarcolemma disruption are the reports of reversible electrophysiological and inotropic effects following exogenous LCACs wash out or after/during normoxic reperfusion post ischemia (Corr et al., 1989; Sakata et al., 1989). LCACs are also proposed to modulate the activity of specific ECC ion channels and enzymes. Similarly, arrhythmogenic effects of exogenous LCACs have been found to be attenuated by pharmacological inhibition of ECC components, as will be discussed below. Whether LCACs directly interact with the ion-handling proteins of cardiac ECC is unclear. Moreover, it is not known whether LCACs indirectly affect ion channel or enzyme function via incorporation into local lipid domains, which could induce a conformational change in the integral membrane proteins.

In sum, the evidence strongly supports membrane perturbation and altered phospholipid packing in mediating the electrophysiological and inotropic effects of LCACs in the heart. However, LCACs also modulate the activity of specific ECC ion channels and enzymes. Currently it is unclear whether direct protein interactions occur to induce these activity modulations, or whether perturbation of local phospholipid domains indirectly changes protein function, which might be dependent on LCAC species, concentration, and exposure time. In the following sections, for clarity LCACs are described as altering specific components of cardiac ECC; however, whether the effects on protein function are direct or indirect is unclear and should be interpreted as such.

#### Ca^2+^-Handling

The key ionic link between cardiomyocyte excitation and contraction is Ca^2+^. Appropriate timing and amplitude of extracellular Ca^2+^ influx and intracellular Ca^2+^ release determines the rhythmicity of activation and the strength of contraction (Bers, 2002). A central mechanistic hypothesis to the effects of LCACs on cardiac function is Ca^2+^ overload within the cardiomyocyte cytosol. This has been shown to be concentration-dependent, as well as consistent across species and myocardial preparations (Clarke et al., 1996; Netticadan et al., 1999).

##### Extracellular Ca^2+^ influx and L-type Ca^2+^ channels

The arrhythmic and positive inotropic manifestations of myocardial LCAC superfusion have been reported to increase in amplitude and frequency proportionally with increases in extracellular Ca^2+^ concentration (Patmore et al., 1989; Sakata et al., 1989; Meszaros and Pappano, 1990). Furthermore, delayed afterdepolarizations and aftercontractions induced by LCACs are prevented in a low (0.1 mM) Ca^2+^ superfusion medium (Sakata et al., 1989). In Fura-2 loaded rat myocytes, the dose-dependent (5–20 μM) increase in cytosolic Ca^2+^ acutely evoked by LCAC superfusion was significantly blunted with EGTA chelation of extracellular Ca^2+^ (Netticadan et al., 1999). These findings demonstrate a dependence on extracellular Ca^2+^. The mechanism of Ca^2+^ entry, however, is less clear.

L-type Ca^2+^ channels (LTCCs) facilitate Ca^2+^ influx during late myocyte depolarization, provoking the Ca^2+^-induced Ca^2+^ release that sustains the action potential plateau and catalyzes myofilament interaction (Bodi et al., 2005). LCAC superfusion has been found to dose- (1–25 μM) and time- (2–10 min) dependently reduce current density, as well as activation and inactivation kinetics of LTCCs (Wu and Corr, 1992; Liu and Rosenberg, 1996; Ziolo et al., 2001). This inhibitory effect occurs regardless of intra- or extracellular LCAC delivery, is completely reversible following wash-out, and is consistent across species and preparations (Wu and Corr, 1992; Liu and Rosenberg, 1996; Ziolo et al., 2001). A biphasic effect of LCACs on LTCC function has been reported, whereby a transient LTCC current and open probability increase occurs at a 1–10 μM dose; however, this progresses to channel inhibition within 3 min of incubation (Liu and Rosenberg, 1996). Paradoxically, LTCC inhibition using verapamil or diltiazem has been found to significantly attenuate LCAC-evoked intracellular Ca^2+^ accumulation (Netticadan et al., 1999). Furthermore, LCAC superfusion acutely reverses the negative inotropic effects of verapamil in a manner resembling that of the LTCC agonist, Bay K 8644, suggesting a stimulatory effect of LCACs on channel function (Patmore et al., 1989). In contrast, others have found no effect of verapamil on *ex vivo* arrhythmias provoked by LCACs despite an inhibition of arrhythmias in low extracellular Ca^2+^ (Sakata et al., 1989). This discrepancy in reports of LTCC dependence in LCAC-provoked Ca^2+^ overload could be due to differences in LCAC and verapamil doses, as well as the myocardial preparation used (i.e., isolated myocyte vs. multicellular preparation).

##### Sarcoplasmic reticulum Ca^2+^ release and uptake

Sarcoplasmic reticulum (SR) Ca^2+^ release and uptake mechanisms facilitate the majority of systolic intracellular Ca^2+^ increases and diastolic decreases in the mammalian myocardium, respectively (Bers, 2002). In cardiac muscle, the primary SR Ca^2+^ release channel is the cardiac ryanodine receptor (RyR2), while Ca^2+^ is re-sequestered by the action of the SR Ca^2+^-ATPase (SERCA2a) (Bers, 2002). LCAC superfusion has been found to affect both RyR2 and SERCA2a.

Recent work by Roussel et al. (2015) showed that 10 μM LCAC superfusion enhanced the rate of cardiomyocyte SR Ca^2+^ leak, resulting in reductions in the SR Ca^2+^ content and cytosolic Ca^2+^ transients. Additionally, this Ca^2+^ leak enhanced spontaneous Ca^2+^ spark and Ca^2+^ wave propensity, which was linked to LCAC-mediated *in vivo* premature ventricular complexes. These findings support earlier reports of rapid, dose-dependently enhanced ^45^Ca^2+^ release from cardiac (Yamada et al., 2000) and skeletal muscle (El-Hayek et al., 1993; Dumonteil et al., 1994) SR vesicles exposed to LCACs. The direct dependence on RyR2-mediated Ca^2+^ release is controversial. Pre-treatment with low micromolar doses (0.1–5 μM) of ryanodine, which depletes SR Ca^2+^ (Meissner, 1986), attenuates the *ex vivo* arrhythmia incidence and cytosolic Ca^2+^ accumulation induced by LCAC superfusion (Sakata et al., 1989; Meszaros and Pappano, 1990; Netticadan et al., 1999). These indirect implications of RyR2-dependence are supported by findings in skeletal muscle SR vesicles showing LCAC-mediated ^45^Ca^2+^ release is partially blocked by co-treatment with the ryanodine receptor inhibitor, ruthenium red (El-Hayek et al., 1993; Dumonteil et al., 1994). Furthermore, LCACs dose-dependently enhance [^3^H]ryanodine binding within the SR membranes and markedly increase skeletal muscle ryanodine receptor (RyR1) open probability (El-Hayek et al., 1993; Dumonteil et al., 1994). In contrast, neither ruthenium red nor high micromolar (100 μM) ryanodine, which inhibits RyR2 function (Meissner, 1986), affected [^3^H]ryanodine binding or the rapid ^45^Ca^2+^ release induced by LCAC treatment (Yamada et al., 2000). Therefore, there is a common dependence on SR Ca^2+^ release in both skeletal and cardiac muscle; however, the direct effect of LCACs on ryanodine receptor activity differs. The effect of LCACs on cardiac RyR2 may instead be indirect via post-translational modification, such as the RyR2 oxidation proposed by Roussel et al. (2015) in their recent work.

Coupled to the SR Ca^2+^ release induced by LCAC treatment is a well-established inhibition of SERCA2a. Monophasic inhibition of SERCA2a activity has been reported in SR vesicles from canine, rabbit, and rat cardiac tissues at a variable concentration range (Pitts et al., 1978; Pitts and Okhuysen, 1984; Dhalla et al., 1991; Yamada et al., 2000). Pitts et al. found a half-maximal SERCA2a inhibition at ∼20 μM LCAC in canine SR (Pitts et al., 1978), which is analogous the dose-dependent (10–50 μM) inhibition reported by Dhalla et al. (1991) and Yamada et al. (2000) in rabbit and rat SR vesicles, respectively. Similarly, Dumonteil et al. (1994) reported inhibition of SERCA1a in skeletal muscle; however, this required a slightly higher LCAC dose range (20–100 μM). Biphasic inhibition of SERCA2a has also been reported. Early work by Adams et al. found that lower LCAC concentrations (5–50 μM) stimulated SERCA2a activity; however, doses ≥ 50 μM induced dose-dependent inhibition of enzyme function (Adams et al., 1979a). In isolated rat myocytes, pharmacological blockade of SERCA2a with thapsigargin was found to significantly attenuate the increase in cytosolic Ca^2+^ concentration induced by LCAC superfusion (Netticadan et al., 1999). Taken together with the dependence on Ca^2+^ release from the SR, regardless of RyR2-dependency, LCACs appear to alter both SR Ca^2+^ uptake and release in a manner that promotes cytosolic Ca^2+^ overload and can be translated to other reports of arrhythmogenic and inotropic effects of LCACs.

##### Mitochondrial Ca^2+^

As in the sarcolemma and cytosol, mitochondrial LCAC content is increased in ischaemia or with exogenous LCAC superfusion (Idell-Wenger et al., 1978; Liedtke et al., 1978; Knabb et al., 1986; McHowat et al., 1993). The increased LCAC content is associated with a dose-dependent (5–50 μM) decrease in mitochondrial Ca^2+^ uptake rate and increase in Ca^2+^ release rate (Wolkowicz and McMillin-Wood, 1980; Piper et al., 1984; De Villiers and Lochner, 1986). The contribution of dysfunctional mitochondrial Ca^2+^ flux to Ca^2+^ overload induced by LCACs has not yet been determined. Work in isolated mitochondria and Langendorff-perfused rat hearts has linked LCAC-mediated dysfunctional mitochondrial Ca^2+^-handling with a decrease in oxidative metabolism and ATP production (Piper et al., 1984; Baydoun et al., 1988; Hara et al., 1997; Xiao et al., 1997; Arakawa and Hara, 1999). Furthermore, inhibition of LCAC generation is reported to prevent metabolic deficits in the ischaemic myocardium in parallel with normalization of myocyte electrophysiology and in vivo function (Lopaschuk et al., 1988; Yamada et al., 1994). These findings suggest a link between mitochondrial Ca^2+^, metabolic inhibition, and cardiac dysfunction during excessive LCAC exposure; however, others have reported that action potential alterations induced by LCACs persist with metabolic blockade (Sakata et al., 1989).

#### Na^+^-Handling

Na^+^-handling is the primary determinant of cardiomyocyte excitability (Bers and Despa, 2009). Depolarization of the myocardium leads to rapid activation and deactivation of voltage-gated Na^+^ channels, which determines the action potential V_*max*_ and APA and initiates downstream Ca^2+^-handling for contraction (Bers and Despa, 2009). Additionally, the sarcolemma Na^+^/Ca^2+^ exchanger (NCX1) contributes to cytosolic Ca^2+^ clearance in diastole, while the ubiquitous Na^+^/K^+^-ATPase (NKA) energetically maintains the stable cardiac RMP (Bers and Despa, 2009). LCACs alter many facets of cardiac Na^+^-handling, centring around an increase in intracellular Na^+^ concentration (Wu and Corr, 1995).

##### Inward Na^+^ current and Na^+^/Ca^2+^ exchange

LCAC superfusion of rodent myocytes induces a dose-dependent inhibition of the early inward Na^+^ current (I_*Na*_), accounting for LCAC-mediated reductions in action potential V_*max*_ and APA (Sato et al., 1992). However, LCACs promote the formation of a tetrodotoxin (TTX)-sensitive late Na^+^ current (I_*Na(L*__)_) that exhibits a markedly prolonged channel inactivation period and drives an increase in intracellular Na^+^ concentration (Wu and Corr, 1994). This I_*Na(L)*_ has been found to occur spontaneously within 2 min of LCAC exposure and to activate a secondary transient inward current (I_*ti*_) (Wu and Corr, 1994, 1995). Intracellular dialysis of EGTA (Ca^2+^ chelator) or extracellular NiCl_2_ (NCX1 inhibitor) application revealed that this I_*ti*_ is dependent on intracellular Ca^2+^ and sarcolemma Na^+^/Ca^2+^-exchange. Furthermore, I_*Na(L)*_ inhibition prevented the formation of I_*ti*_ and spontaneous afterdepolarizations induced by LCACs, thereby validating the temporal association of Na^+^ accumulation with Na^+^/Ca^2+^-exchange and Ca^2+^ overload in arrhythmogenesis (Wu and Corr, 1994, 1995). This is supported by the finding that myocyte Ca^2+^ accumulation evoked by LCAC superfusion is augmented in low extracellular Na^+^ media, while overload does not occur with NCX1 inhibitors, NiCl_2_ or amiloride (Netticadan et al., 1999). Besides the implication of the I_*Na(L)*_ in LCAC-mediated arrhythmias, treatment with I_*Na(L)*_ inhibitors lidocaine, ranolazine, or TTX prevents LV diastolic dysfunction in Langendorff rat hearts (Arakawa et al., 1997; Maruyama et al., 2000; Wu et al., 2009). Blockade of LCAC-mediated I_*Na(L)*_ is also suggested to reduce the metabolic deficit developed with LCAC treatment (Arakawa et al., 1997; Maruyama et al., 2000; Wu et al., 2009). Moreover, ranolazine has been found to prevent LCAC-dependent upregulation of α-adrenergic receptor expression during early ischemia (Heathers et al., 1987; Allely et al., 1993).

##### Na^+^/K^+^-ATPase

Near consensus exists in the literature regarding LCAC inhibition of the sarcolemma NKA. Studies using partially purified canine and bovine NKA (Wood et al., 1977; Pitts et al., 1978; Adams et al., 1979a, b), sarcolemma vesicles (Abe et al., 1984; Kramer and Weglicki, 1985; Dhalla et al., 1991), and isolated myocytes (Tanaka et al., 1992; Wu and Corr, 1995; Shen and Pappano, 1997) have reported an acute enzyme inhibition and reduced [^3^H]ouabain binding with LCAC exposure. This inhibition exhibits dose-dependency ranging from 2.5 up to 600 μM for onset of inhibition – the wide variation likely due to preparation differences and the inter-relatedness of NKA activity with other ion-handling proteins (Pitts and Okhuysen, 1984; Kramer and Weglicki, 1985; Tanaka et al., 1992). Like that associated with blockade of Ca^2+^ and Na^+^ influx, inhibition of NKA with ouabain also reduces the extent of cytosolic Ca^2+^ accumulation (Netticadan et al., 1999). Functionally, many have reported that the effect of LCACs on cardiomyocyte RMP depolarization and positive inotropy is analogous to that of ouabain (Tanaka et al., 1992; Shen and Pappano, 1995; Wu and Corr, 1995; Shen and Pappano, 1997). In contrast, when NKA activity was isolated from that of inward Ca^2+^ an Na^+^ currents, as well NCX1 and the inward rectifying K^+^ current, Wu and Corr (1995) found that LCAC application only modestly depolarises the RMP when compared to ouabain (13 vs. 91% depolarization), suggesting a relatively minor role of the pump in the Na^+^ accumulation (and subsequent Ca^2+^ overload) induced by LCACs.

#### K^+^-Handling

Cardiac K^+^-handling controls action potential repolarization kinetics and maintenance of resting electrochemical gradients; therefore, modulations of K^+^-handling predominantly alter APD and the RMP (Schmitt et al., 2014; Grandi et al., 2017). In conjunction with inhibition of NKA activity, LCACs have also been reported to alter specific K^+^ currents.

The inward-rectifier K^+^ current (*I*_*K1*_) is tightly coupled with the NKA in the polarization of the RMP (Grandi et al., 2017). Some have reported an inhibition of *I*_*K1*_ in guinea pig myocytes following LCAC exposure at a concentration range of 10–50 μM (Sato et al., 1993; Shen and Pappano, 1997). This inhibition was associated with a depolarization of the RMP and afterdepolarizations (Sato et al., 1993; Shen and Pappano, 1997), fitting within the well-established electrophysiological effects of LCAC superfusion. Others, however, found no effect of either intra- or extracellular LCAC delivery on *I*_*K1*_ or channel kinetics at a similar LCAC dose range (Xu and Rozanski, 1998; Ferro et al., 2012). Furthermore, when isolated from other LCAC-modulated K^+^, Ca^2+^, and Na^+^ currents, the suppression of *I*_*K1*_ was found to contribute to less to RMP depolarization than that of LCAC-mediated NKA inhibition (Shen and Pappano, 1997).

Another K^+^-mediated electrophysiological derangement induced by LCAC application is a reduction of the APD. Cardiomyocyte repolarization, which is the primary determinant of APD, is dictated by a series of voltage- and ligand-gated K^+^ channels (Schmitt et al., 2014; Grandi et al., 2017). Recent work by Ferro et al. demonstrated that LCAC superfusion of a recombinant HEK-293 cell model induces a dose-dependent (1–30 μM) increase in hERG current (*I*_*hERG*_) amplitude (responsible for rapid delayed rectifier K^+^ current). This was associated with an enhancement in deactivation kinetics that, when fitted to computational models, was consistent with an attenuated APD. Furthermore, the authors reported subtle differences in *I*_*hERG*_ stimulation depending on LCAC chain length (Ferro et al., 2012). In contrast, Wu et al. found that pharmacological *I*_*hERG*_ blockade in Langendorff-perfused guinea pig hearts failed to prevent LCAC-dependent mechanical dysfunction (Wu et al., 2009). LCACs have also been suggested to have dose-dependent inhibitory effects on cardiac K^+^ currents, including the transient outward K^+^ current (*I*_*to*_) (Xu and Rozanski, 1998) and ATP-sensitive K^+^ current (*I*_*K,ATP*_) (Haruna et al., 2000), which translate to a prolongation of the APD. However, in one case this was only found if the LCACs were dialysed intracellularly (Xu and Rozanski, 1998) and, in the other case, was closely linked to an interaction of *I*_*K,ATP*_ lipid gating (Haruna et al., 2000).

#### Summary of LCAC Effects on Cardiac Excitation-Contraction Coupling

The electrical and mechanical alterations to cardiac muscle induced by LCAC exposure can be attributed to a pleiotropy of modulations in ECC mechanisms. LCACs inhibit the NKA and promote an *I*_*Na(L)*_, thereby increasing the intracellular Na^+^ concentration and driving arrhythmogenic Na^+^/Ca^2+^ exchange (Wu and Corr, 1994, 1995). This inhibited Na^+^-extrusion is coupled with increases in trans-sarcolemma Ca^2+^ influx, SR Ca^2+^ release, mitochondrial Ca^2+^ efflux, and SERCA2a inhibition that overload cytosolic Ca^2+^ levels. Furthermore, LCACs can alter cardiac K^+^-handling in a manner that would retard the APD (Ferro et al., 2012). With each LCAC-dependent alteration in ECC there is a concentration- and time-dependency, as well as a reversibility of effects. Therefore, LCACs unequivocally induce alterations in various cardiac ECC processes that fit within the framework of arrhythmogenesis and inotropic modulations. Among other limitations, there remain discrepancies in the effect of LCACs on the activity of certain Na^+^, Ca^2+^, and K^+^-handling proteins. Whether LCACs alter these cardiac ECC mechanisms via direct protein interactions, indirect local lipid domain alteration, or through non-specific membrane perturbations remains unclear and requires further investigation. Similarly, the role of downstream LCAC effects, such as reactive oxygen species production, have been implicated in LCAC-mediated alterations of cardiac ECC and, therefore, also warrant further study.

### Research Gaps and Outlook

The purpose of this review was to revisit age-old literature and provide a pathophysiological context to a growing body of metabolomic association studies. The extensive body of basic research and the growing metabolomic associations present LCACs as a therapeutic target in CVD. The efficacy of this therapeutic strategy has previously been explored. Findings from early translational studies showed that the CPT I inhibitor, Etomoxir, had a cardioprotective role in the ischaemic rat heart by decreasing cellular LCAC content and promoting glycolytic substrate utilization (Lopaschuk et al., 1988). Similarly, long-term Etomoxir treatment was found to protect against myocardial hypertrophy and negative inotropy caused by pressure-overload in rat hearts (Turcani and Rupp, 1997). When combined with the attenuation of acute LCAC-induced electrophysiological derangements associated with POCA inhibition of CPT I (Corr et al., 1989), clinical trial of commercial CPT I inhibitors commenced. Long-term Etomoxir treatment improved left ventricular ejection fraction of congestive heart failure patients, although no placebo control was performed (Schmidt-Schweda and Holubarsch, 2000). Randomized controlled trial of Perhexiline, which inhibits both CPT I and CPT II, also reported improvements in ventricular function, as well as in quality of life, thereby supporting CPT enzyme modulation as a novel therapy for CVD (Lee et al., 2005). Critically, however, further clinical trials of CPT inhibition were discontinued due to a key limitation of targeting this enzyme and LCACs: off-target effects (Holubarsch et al., 2007). Due to the ubiquitous nature of LCACs as intermediates of fatty acid metabolism, cardiac-specific targeting of LCACs remains a challenge. Furthermore, as presented above, the basic research indicates that LCACs can alter many facets of cardiac ECC, several of which remain controversial and require further investigation. The identification of circulating LCAC profiles in CVD, in combination with the growing use of metabolomics in the clinic, suggest that the outlook for LCACs in CVD research and medicine in the near future may instead be in a diagnostic and prognostic capacity.

#### Limitations in the Literature

A limitation of the LCAC literature is that all research was performed using non-human models of the mammalian myocardium. Exogenous LCACs affect multiple proteins and mechanisms of cardiac ECC, many of which are differentially expressed between animal models and/or function with a divergent ionic stoichiometry (Bers, 2002; Milani-Nejad and Janssen, 2014). Such differences alter the dynamics of ion homeostasis, rendering species-specificities in electrophysiology, inotropic responses to stimuli, and arrhythmia susceptibility that limit the translatability of the findings (Milani-Nejad and Janssen, 2014). Similarly, there is a dearth of investigation into the effects of LCACs on atrial myocardium, which has significantly different Ca^2+^ propagation, membrane distribution, and re-entry susceptibility when compared to ventricular myocardium (Gaborit et al., 2007; Grandi et al., 2011; Heijman et al., 2016). In addition to species and heart chamber-specific limitations, ECC protein expression patterns are affected by diseases and disorders that closely linked to CVD, such as type 2 diabetes. In fact, chamber-specific Ca^2+^-handling protein expression can even be differentially affected in type 2 diabetes (Bussey et al., 2015). Several species of LCAC are upregulated in the plasma of pre-diabetic and type 2 diabetic individuals relative to match non-diabetics (Adams et al., 2009; Mai et al., 2013). Therefore, as circulating LCACs have been independently associated with CVDs and type 2 diabetes, the intersection of these two associations with the pathogenesis and progression of diabetic cardiomyopathies is currently unknown. Because metabolic remodeling is a hallmark of HF (Ingwall, 2009) and atrial fibrillation (Harada et al., 2017) it is pertinent to determine if insulin resistance and diabetes alters how LCACs are handled in these hearts and whether this cardio-metabolic interaction drives disease phenotype. A further limitation, which has been widely acknowledged (Busselen et al., 1988; Yamada et al., 2000), is that exogenous LCAC effects are more correctly estimated by determining the LCAC to protein or membrane mole ratios instead of utilizing absolute LCAC concentrations. In fact, administering accurate doses of LCAC to superfusion media is difficult due to the tendency of amphiphilic metabolites to segregate at the air/water interface (Piper et al., 1984; Busselen et al., 1988). Furthermore, the critical micelle concentration of LCACs varies significantly based on fatty acyl chain length, the saturation status, as well as the temperature and ionic components of the superfusion solution (Piper et al., 1984). Therefore, determining what is a “pathological” dose of a specific LCAC will vary depending on the membrane distribution and integrity of the myocardial preparation, the experimental conditions, as well as the relative incorporation and/or cellular penetration of the superfused LCAC (Piper et al., 1984). As amphiphilic compounds, LCAC transport within the plasma will be via carrier proteins, like albumin. Interestingly, use of an extracellular carrier protein for exogenously applied LCAC has not been utilized in experimental studies. Therefore, the discrepancy in inotropic effect of LCACs cannot be attributed to the relative presence of “free” vs. “bound” LCACs within the perfusion buffer. Regardless, the absence of albumin in the experimental system limits the translatability to how LCACs may be delivered to the myocardium *in vivo*. Much of the literature has focussed on the myocardial action of one LCAC species, palmitoylcarnitine (LCAC 16:0), due to its relative abundance in the ischaemic myocardium (Idell-Wenger et al., 1978). In turn, the concentration of exogenous palmitoylcarnitine used in many studies is based on measurements of total LCAC content, rather than that of the specific LCAC species. Evidence suggests there might be differences in ECC mechanism alterations between LCAC species (Ferro et al., 2012); therefore, care should be taken to utilize concentrations associated with specific LCAC species, rather than LCACs as a general metabolite class.

#### Future Research Perspectives

In accordance with these limitations, many prospects for future basic research arise that could enhance the translation to metabolomic CVD associations. Firstly, studies utilizing human myocardium should be performed in conjunction with metabolomic investigations. This could be in the form of trabeculae from atrial appendages of cardiac surgery patients or myocardial preparations isolated from transplanted human hearts. Furthermore, studies in human atrial- and ventricular-programmed induced pluripotent stem cells or engineered heart tissues will allow for more long-term studies and higher throughput in elucidating LCAC pathophysiology and potential targeting strategies. Secondly, continued investigation of effects attributable to specific LCACs is required for understanding the pathophysiological aetiologies of arrhythmias and cardiomyopathies associated with LCACs. In the clinic, this could translate to patient-specific CVD diagnosis and risk stratification based on an individual’s metabolomic profile. Moreover, because circulating LCAC levels often correlate with disease severity, NYHA class, and are reduced by surgical intervention, continued monitoring of a CVD patient’s metabolome could provide insight into the progression or regression of the disease (Ahmad et al., 2016; Hunter et al., 2016; Lanfear et al., 2017; Ruiz et al., 2017; Elmariah et al., 2018; Verdonschot et al., 2019). Thirdly, as described above, there is conflicting evidence regarding the dependence on modulations of cardiac metabolism with LCAC treatment. Some that LCAC-induced cardiomyocyte arrhythmias persist despite blockade of glycolysis and oxidative metabolism (Sakata et al., 1989). Others, by contrast, report reductions in mitochondrial ATP production following LCAC treatment, as well as acute increases in mitochondrial reactive oxygen species (Piper et al., 1984; Xiao et al., 1997; Roussel et al., 2015). Given the role of mitochondrial dysfunction and metabolic remodeling in CVD etiology (Zhou and Tian, 2018), translating high circulating LCACs to CVD pathophysiology will require elucidation of LCAC effects on mitochondrial health and myocardial metabolism. Finally, there has been a growing interest in cellular signaling mechanisms altered by acute and chronic exposure to LCACs and Ca^2+^ overload mediated by the acylcarnitines [reviewed by McCoin et al. (2015a)]. Much of this research has been in the context of CVD-adjacent metabolic diseases; however, relating how LCACs modulate inflammatory and apoptotic signaling, as well as kinase activities, could expand the understanding of how LCACs affect cardiac contractility, electrophysiology, and arrhythmias (Rutkowsky et al., 2014; McCoin et al., 2015b).

Besides CVD, developing a greater understanding of cellular LCAC pathogenesis will be informative for the disease processes of rare, but often lethal, fatty acid oxidation and carnitine metabolism disorders (Merritt et al., 2018). Metabolomic profiling has been routinely used in diagnosis of these disorders for many years, with specific metabolite upregulations indicating which metabolic component is defective (McHugh et al., 2011). Disease management commonly involves reductions in dietary fat intake and avoidance of fasting; however, relatively little is known about the cellular processes underpinning the disease symptoms (Spiekerkoetter et al., 2009; Merritt et al., 2018). Many of these metabolic disorders are associated with cardiovascular complications (among many systemic symptoms), including severe cardiomyopathies and fatal arrhythmias (Isackson et al., 2008; Merritt et al., 2018). Therefore, an enhanced understanding of the mechanisms mediating LCAC driven electrophysiological derangements and inotropic modulations, as well as those attributable to different LCAC species, could offer therapeutic targets during acute cardiac events caused by the metabolic disorders. From an alternative perspective, knowledge of symptom management by dietary modification acquired from fat metabolism disorders could be informative if control of circulating LCAC levels was used as a strategy in CVD management. This strategy would align with recent longitudinal study that identified a link between 15 year CVD risk, dietary intake, and the associated modulations in the metabolomic profile (Akbaraly et al., 2018). Furthermore, it supports recent postulation within the cardiovascular metabolism field that monitoring and re-balancing of myocardial metabolism is a promising interventional strategy in cardio-metabolic disease (Glatz et al., 2020).

## Conclusion

This review addressed the existing basic science investigations into the direct effects of the amphiphilic metabolites, LCACs, on cardiac function. LCACs induce a pleiotropy of effects on key ion-handling processes within the mammalian myocardium, manifesting in stereotypical alterations to the cardiac action potential that enhance the propensity for arrhythmic muscle activity. These metabolites also alter myocardial contractility; however, whether the effect is positively or negatively inotropic is inconclusive. Despite an extensive body of work establishing LCACs as effectors of cardiac muscle, several key limitations exist that form an incomplete understanding of the role of LCACs on the human heart. The evidence presented highlights how these intermediates of metabolism can affect the function of the ischaemic and non-ischaemic myocardium and provides a pathophysiological context to the growing body of metabolomic implications of LCACs in CVD.

## Author Contributions

HA-B reviewed the literature. HA-B and RL wrote the first version of the manuscript and prepared the figures. JK, TZ, and PJ reviewed and edited the manuscript. All authors read and approved the final manuscript.

## Conflict of Interest

The authors declare that the research was conducted in the absence of any commercial or financial relationships that could be construed as a potential conflict of interest.
